# A Highly Reliable, 5.8 GHz DSRC Wake-Up Receiver with an Intelligent Digital Controller for an ETC System

**DOI:** 10.3390/s20144012

**Published:** 2020-07-19

**Authors:** Imran Ali, Muhammad Asif, Muhammad Riaz Ur Rehman, Danial Khan, Huo Yingge, Sung Jin Kim, YoungGun Pu, Sang-Sun Yoo, Kang-Yoon Lee

**Affiliations:** College of Information and Communication Engineering, Sungkyunkwan University (SKKU), Suwon 16419, Korea; imran.ali@skku.edu (I.A.); m.asif@skku.edu (M.A.); riaz@skku.edu (M.R.U.R.); danialkhan@skku.edu (D.K.); yingge@skku.edu (H.Y.); sun107ksj@skku.edu (S.J.K.); hara1015@skku.edu (Y.P.); rapter@kaist.ac.kr (S.-S.Y.)

**Keywords:** wake-up receiver, digital controller, reliability, electronic toll collection (ETC) system, dedicated short range communication (DSRC)

## Abstract

In this article, a highly reliable radio frequency (RF) wake-up receiver (WuRx) is presented for electronic toll collection (ETC) applications. An intelligent digital controller (IDC) is proposed as the final stage for improving WuRx reliability and replacing complex analog blocks. With IDC, high reliability and accuracy are achieved by sensing and ensuring the successive, configurable number of wake-up signal cycles before enabling power-hungry RF transceiver. The IDC and range communication (RC) oscillator current consumption is reduced by a presented self-hibernation technique during the non-wake-up period. For accommodating wake-up signal frequency variation and enhancing WuRx accuracy, a digital hysteresis is incorporated. To avoid uncertain conditions during poor and false wake-up, a watch-dog timer for IDC self-recovery is integrated. During wake-up, the digital controller consumes 34.62 nW power and draws 38.47 nA current from a 0.9 V supply. In self-hibernation mode, its current reduces to 9.7 nA. It is fully synthesizable and needs 809 gates for its implementation in a 130 nm CMOS process with a 94 × 82 µm^2^ area. The WuRx measured power consumption is 2.48 µW, has −46 dBm sensitivity, and a 0.484 mm² chip area.

## 1. Introduction

**Figure 1:**
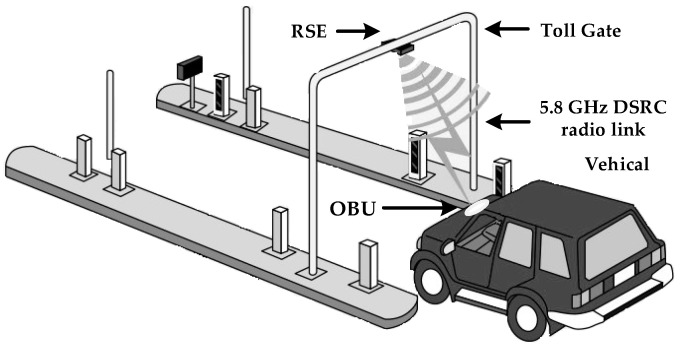
The dedicated short range communication (DSRC) system overview with road side equipment (RSE) fitted at toll gate, battery operated on-board unit (OBU) fixed inside the vehicle, and a 5.8 GHz DSRC radio link as communication channel.

**Figure 2:**
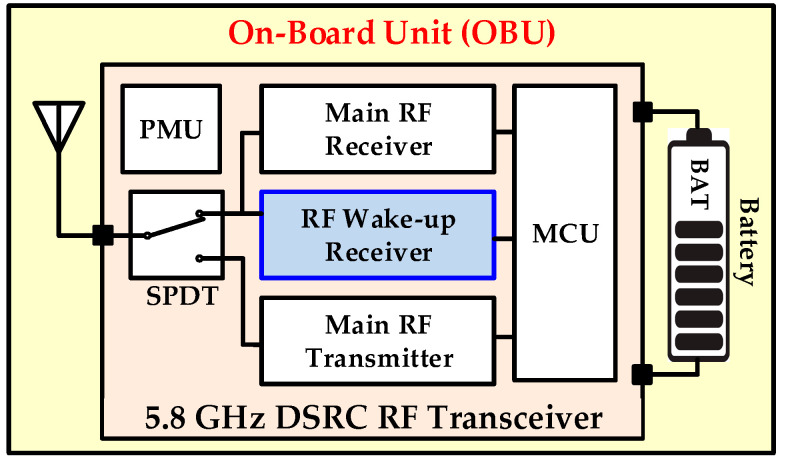
The DSRC OBU system level block diagram.

**Figure 3:**
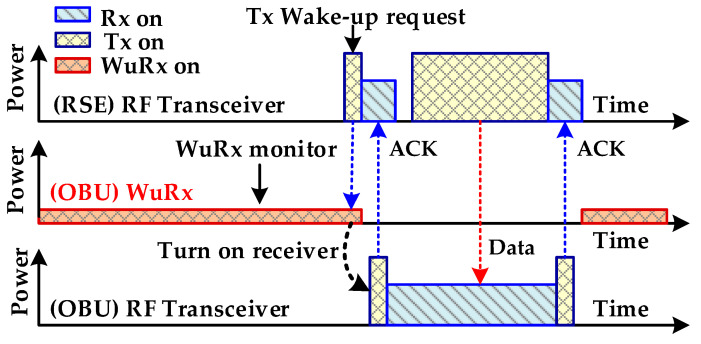
The road side equipment (RSE) and OBU pure asynchronous communication with the RF wake-up receiver (WuRx).

**Figure 4:**
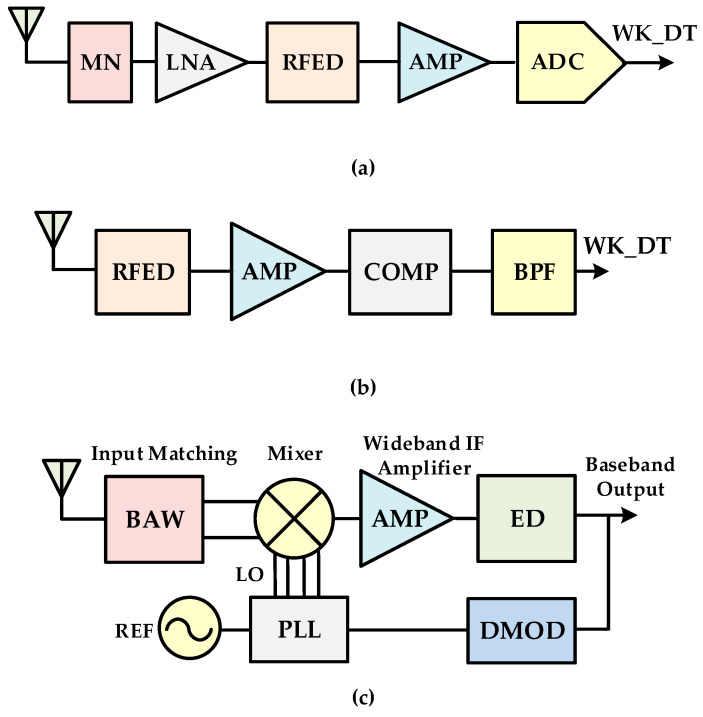

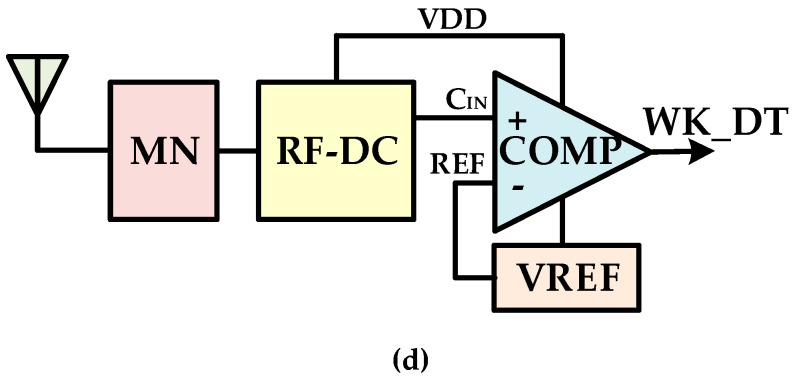
The previous wake-up receiver architecture: (**a**) RF envelope detector (RFED) based with analog to digital converter (ADC); (**b**) RFED based with band pass filter (BPF); (**c**) frequency conversion with LO; (**d**) passive circuit with RF-DC.

Recently, the radio frequency (RF) wake-up receiver has become an attractive research area for battery-operated transceivers in a variety of applications such as electronic toll collection (ETC) systems, wireless sensor networks (WSNs), wireless body area networks (WBANs), internet-of-things (IoTs), and wearable devices [1,2,3,4]. Nowadays, the ETC system (ETCS) is rapidly being adopted as an intelligent transportation solution in automotive vehicles. It uses a 5.8-GHz dedicated short range communication (DSRC) for a high speed radio link between a road side equipment (RSE) fixed at the toll gate and on-board unit (OBU) fitted inside the vehicle [1,5,6,7], as shown in Figure 1. Without stopping the vehicles, the toll is paid automatically and it saves time and eliminates traffic congestion on the roads. In ETCS, a wake-up receiver (WuRx) is an auxiliary RF receiver, additionally to the main RF transceiver, as shown in Figure 2, and is mandated due to the battery powered OBU. The WuRx is a pure asynchronous communication scheme and it maximizes data transceiver sleep time. This not only reduces OBU energy dissipation but also diminishes network latency. Figure 3 shows the asynchronous communication between RSE and OBU with WuRx. The reliability, false wake-up, power dissipation, and sensitivity are key considerations in WuRx design. In WuRx, designed with low power dissipation and good sensitivity, false and poor wake-up turns on main transmitter and receiver modules, which reduces battery life and degrades the overall WuRx performance. The numerous WuRx circuits have been investigated in literature to optimize power consumption, maximize sensitivity, and improve reliability [8,9,10,11]. The various WuRx architectures are summarized in Figure 4. Based on the type of the power source, the WuRx is categorized in active and passive wake-up circuits. The active WuRx circuits are powered from a battery fitted on an OBU to monitor a possible wake-up signal. The energy for the wake-up circuit is harvested from the incident RF signal in passive receivers. Mostly, the active circuits adopt either RF envelope detector (RFED) structures [1,12,13], shown in Figure 4a,b, or frequency conversion architectures [14,15], depicted in Figure 4c. The frequency conversion based wake-up receivers offer higher sensitivity due to RF amplification before RF envelope detection [16], or local oscillator (LO) generation for down-conversion before amplification and envelope detection at intermediate frequency (IF) [14]. These circuits dissipate more power and occupy a larger area due to a power hungry phase-locked loop (PLL) and automatic channel scanning circuits for two channel receptions [15]. The majority of wake-up circuits are implemented with RFED because of its low power consumption using Schottky diodes [12,13] or metal–oxide–semiconductor field-effect transistor (MOSFET) operating in the weak-inversion region. The WuRx structure shown in Figure 4a uses an analog to digital converter (ADC) after a programmable gain amplifier (PGA) which occupies more area and increases power consumption. The WuRx shown in Figure 4b uses an analog band pass filter (BPF) at the output interface which requires more chip area. Figure 4c shows an envelope detector based WuRx structure with a front end amplifier and bulk acoustic wave (BAV) input network [17,18]. The WuRx in [19] also incorporates ADC at the output; however, in order to achieve better sensitivity and to reduce receiver noise, it incorporates a low noise amplifier (LNA) before an envelope detector and uses a double sampling technique. It offers better sensitivity at the cost of increased power consumption and larger chip area for WuRx. The passive WuRx structure [20] incorporates radio frequency to direct current (RF-DC) converter for harvesting energy from incident RF signals as shown in Figure 4d. For this purpose, an RF-DC converter [21,22] is employed to produce the envelope of the on–off keying (OOK) wake-up message signal, and at the same time, it efficiently converts the RF carrier to a DC voltage in order to supply the comparator and the other WuRx circuits. Although this architecture is power efficient, it exhibits low sensitivity. Furthermore, the deficiency of false and poor wake-up filtering is vulnerable. 

The wake-up signal is categorized as a single wake-up tone of a bit sequence [23]. The bit sequence signal, also called identity-based wake-up, is widely used in WSN and WBAN for addressing a particular destination sensor node for unicasting. For broadcasting, single wake-up tone is adopted [23]. The DSRC wake-up signal is a 14 kHz OOK single tone signal of 15–17 cycles which is modulated with a 5.8 GHz carrier frequency [9].

The precise sensitivity control is essential for ETCS wake-up circuits [1]. Neither a very low nor very high sensitivity is intended. The available communication time will be insufficient when WuRx sensitivity is lower than a minimum level. Similarly, if WuRx has very high sensitivity, it will turn on OBU and start communication even if vehicle is far away from a RSE and toll area. Moreover, very high sensitivity will result in communication failure and interrupts other OBU devices on the road. Therefore, WuRx sensitivity must be in a range, robust, insensitive to process, voltage, and temperature (PVT) variations [11].

The key characteristic of WuRx is to detect and ensure the presence of wake-up request in the received RF signal and decide whether to turn on the power hungry transceiver on OBU. In most previous studies, the WuRx circuits only identify the signal amplitude which is inadequate in ETC systems. If false and poor wake-up signals are not identified and filtered out in the WuRx circuit, these signals turn on power hungry main RF transceiver modules and the battery performance is degraded. In the past, numerous WuRx architectures have been investigated for improving the sensitivity and reducing the power consumption. However, false and poor wake-up problems have been left unaddressed. This paper presents a RFED based highly reliable WuRx. An intelligent digital controller (IDC) is proposed to ensure the WuRx reliability and accuracy by identifying and rejecting unwanted, false, and poor wake-up signals. It also replaces complex blocks such as ADC and BPF after amplifier and comparator [8,9,10,24] and reduces current consumption and area. Due to its digital nature, it is fully synthesizable, immune to noise and PVT variations [25], offers system flexibility, a wide dynamic range for wake-up and oscillator frequencies, and is adaptive to technology scaling.

The rest of the paper is organized as follows: Section 2 presents a proposed WuRx architecture overview. The detailed design of the proposed digital controller is described in Section 3. The tunable range communication (RC) oscillator is presented in Section 4. The RF front end and baseband analog processing is included in Section 5. Section 6 describes the experimental results. Lastly, the paper is concluded in Section 7. 

## 2. Proposed Wake-Up Receiver Architecture

**Figure 5:**
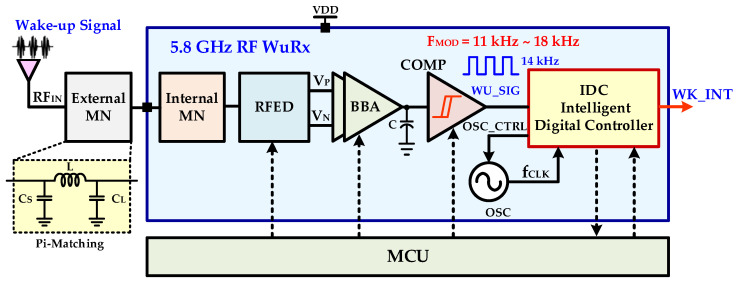
Proposed RF wake-up receiver architecture.

Figure 5 shows the proposed 5.8-GHz RF WuRx in which IDC is integrated to ensure its reliability and accuracy. An antenna receives the incoming RF signal and sends it to an off-chip pi-matching network. The matching network is an essential passive circuit to transfer the maximum RF signal power to the receiver circuit. The pi-matching network matches the antenna equivalent impedance with the input impedance of the proposed WuRx and ensures the maximum power transfer from the antenna to the WuRx circuit. Unlike [8,24], the chip internal matching network in addition to off-chip pi-matching receives a RF wake-up signal, boosts voltage, and improves sensitivity and |*S*_11_|. The high gain RF envelope detector recovers the baseband wake-up signal and improves signal-to-noise ratio (SNR) without additional current consumption. The RFED is a critical circuit and it interfaces WuRx with the antenna, down-converts the amplitude of the modulated RF 5.8 GHz signal, and generates a 14 kHz baseband wake-up signal. The PGA provides flexibility to improve gain and amplify the baseband signal significantly. The main control unit (MCU) enables/disables and configures different programmable parameters of RFED, baseband analog (BBA), comparator (COMP), and IDC. It can either be an on/off chip modem, externally controllable registers, or an external microcontroller. The comparator (COMP) generates digital output for IDC processing. An ultra-low power range communication (RC) oscillator (OSC) with a dynamic tuning range generates a configurable clock for the IDC block. The digital controller is proposed for ensuring WuRx reliability and accuracy by identifying and filtering non-wake-up signals. It is a fully synthesizable block, consumes very low power, and needs a very small chip area. The digital controller also replaces complex power consuming and large area interface blocks, such as ADC, BPF.

## 3. Multi-Mode, Configurable Intelligent Digital Controller (IDC)

**Figure 6:**
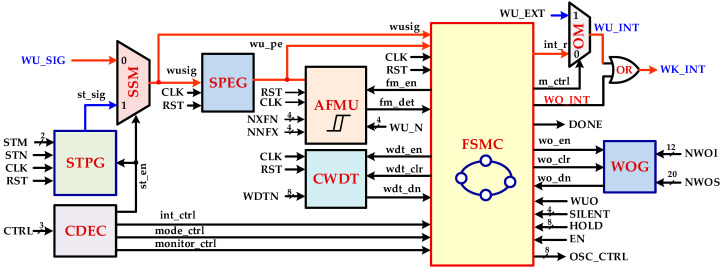
RF WuRx intelligent digital controller (IDC) architecture.

**Figure 7:**
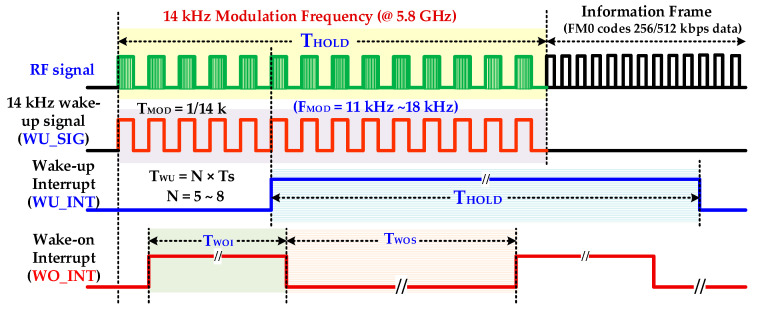
RF wake-up and wake-on interrupt timing diagram.

In the DSRC WuRx design, other than high sensitivity and low power consumption, the false and poor wake-up signals identification is very crucial in order to extend OBU battery lifetime. This is achieved by filtering non-wake-up and noise signals and prohibiting turning on main power hungry RF transceiver. For this purpose, a novel, multi-mode, and configurable intelligent digital controller is proposed for ensuring WuRx reliability and accuracy. This controller is also a low power, small area digital replacement of complex, high power analog blocks such as ADC and a band pass filter. Different parameters of wake-up, self-hibernation, digital hysteresis, wake-on, watch-dog timer, and self-test are fully configurable, which make the controller architecture very flexible and adaptive. The simplified architecture of a WuRx digital controller is illustrated in Figure 6 and the timing diagram is elaborated in Figure 7 for DSRC applications. The signal selection multiplexer (SSM) selects either a baseband wake-up signal, *WU_SIG*, from the comparator output in normal operation or self-test signal *st_sig*, generated from self-test pattern generator (STPG) during test mode. The signal positive edge generator (SPEG) detects rising transitions in the final selected signal *wusig* and generates a pulse signal *wu_pe.* The finite state machine controller (FSMC) is the key building block of IDC which is designed as control unit and data path. It mainly senses, ensures, and generates the wake-up interrupt and filters unwanted signals. It controls other blocks such as the adaptive frequency measurement unit (AFMU), configurable watch-dog timer (CWDT), and wake-on generator (WOG). When enabled by FSMC (*fm_en* = 1), the AFMU measures the frequency of the wake-up signal and determines by generating signal *fm_det* if the input signal value is either within the configured range or not. It also ensures the valid successive number of configured (*WU_N*) wake-up signal cycles. The CWDT, when enabled by signal *wdt_en* from FSMC, starts a timer. The timer duration is configurable from the *WDTN* parameter. It enhances IDC reliability and helps to avoid any halt situation during frequency measuring and signal ensuring states. If there is any abnormal situation, CWDT resets FSMC to its initial state when the configured timer expires. The WOG implements a pseudo-synchronous interrupt generation. When it is enabled, the wake-on interrupt WO_INT is generated instead of the wake-up interrupt. The STPG enhances WuRx reliability by verifying IDC operation in self-test mode. When enabled (*st_en* = 1), it is capable of generating a variety of valid and invalid signals with different frequencies and number of cycles. The output multiplexer (OM) outputs *WU_INT* by selecting either internally generated interrupt signal *int_r* or external manually control interrupt WU_EXT. The control decoder (CDEC) decodes interrupt *int_ctrl*, mode *mode_ctrl*, and monitor *monitor_ctrl* control signals from the external CTRL input.

### 3.1. Self-Hibernation for Low Power Consumption

A self-hibernation methodology with dynamic frequency and voltage scaling is introduced for reducing the IDC and oscillator power consumption during the non-wake-up interval. The average power consumption *P_AV_* in the CMOS circuit is the sum of dynamic *P_DYN_*, short circuit *P_SHORT_*, leakage *P_LEAKAGE_*, and static *P_STATIC_* power consumptions [26] as explained in (1) as follows:

(1) $$P_{AV}=P_{DYN}+P_{SHORT}+P_{LEAKAGE}+P_{STATIC}.$$

In CMOS circuits, *P_DYN_* is the dominant power consumption component. It is the linear function of operating frequency *f* and the quadratic function of the supply voltage *V_DD_* of the circuit as given in (2) as follows:

(2) $$P_{DYN}=KCfV_{DD}^{2},$$

where *K* is switching activity factor, *C* is loading capacitance, and *V_DD_* is supply voltage [26]. 

From Equation (2) it becomes obvious that if the switching frequency reduces, the power consumption of the CMOS circuit is reduced significantly. Furthermore, if the supply voltage is minimized, the dynamic power is reduced. The OSC frequency is configurable from IDC. The IDC programs the OSC at a relatively higher frequency, *f_WU_*, and performs WuRx signal identification during the active interval. After the wake-up interval, it operates at a relatively very low frequency, *f_SH_*, during the sleep period after configuring the OSC for the slowest frequency. Since the communication between RSE and OBU is only for a very short duration and the OBU is in sleep mode for most of the time, the self-hibernation proves its significant impact for reducing WuRx power consumption and extending battery lifetime. The supply voltage is also reduced from 1.2 V to 0.9 V to save battery power.

### 3.2. Built-In Self-Test for IDC Reliability 

For ensuring IDC reliability and accuracy, a built-in self-test technique is integrated. It verifies the IDC full operation and functional accuracy without the presence of an external RF wake-up signal. In the presented self-test scheme, a configurable self-test pattern generator module generates a variety of configurable wide range valid wake-up signals with a frequency described as follows in Equation (3):

(3) $$f_{ST}=\frac{N_{ST}}{f_{WU}},$$

where *f_ST_* is test wake-up signal *st_sig* frequency, which is programmable from the *N_ST_* parameter. It is also capable of generating non-wake-up, false and poor wake-up, and noise signals during test mode and guarantees IDC functional accuracy and enhances overall WuRx reliability.

### 3.3. Configurable Modes

In the proposed IDC structure, two fully configurable modes are explored for WuRx. The wake-up mode (WUM) is for purely asynchronous wake-up signal detection from RSE with reduced latency. It processes the baseband recovered signal from the envelope detector after the comparator for identifying the wake-up signal. On the other hand, the wake-on mode (WOM) is an auxiliary pseudo-synchronous mode. When it is enabled, it turns on the main receiver for a very small configurable time interval, listens to any possible request from RSE, and keeps off for a relatively long duration. The mode control signal wake-up/-on (WUO) chooses the current selected mode. When WUO is low, a wake-up mode is enabled, which is the default mode. For enabling WOM, the WUO is configured as high.

#### 3.3.1. Wake-Up Mode

**Figure 8:**
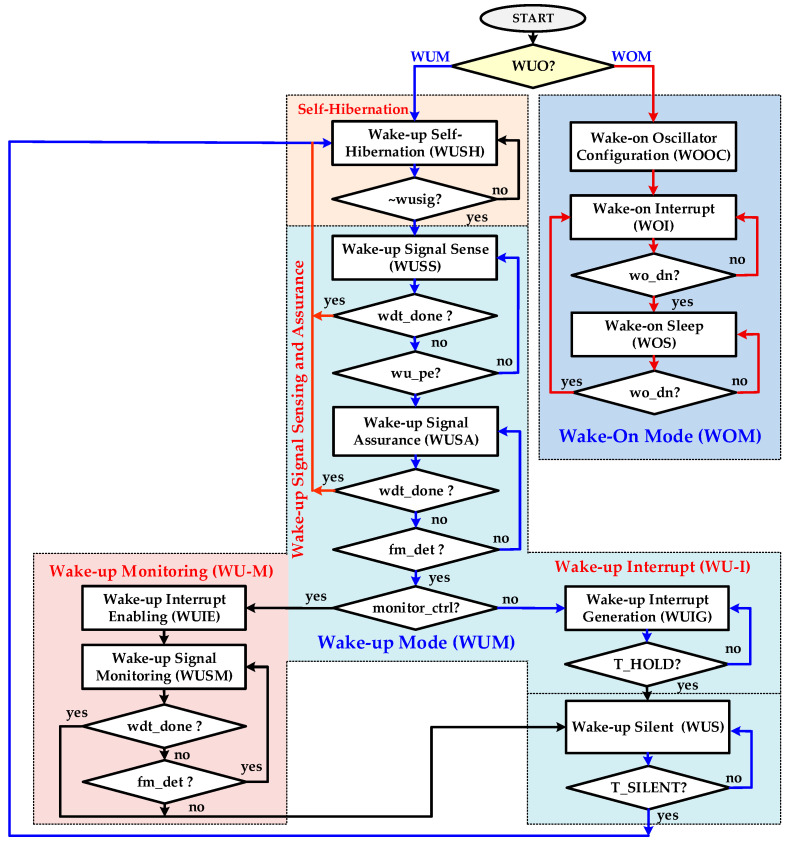
WuRx intelligent digital controller (IDC) finite state machine controller (FSMC) flow diagram.

The FSMC is key building block of IDC, and its flow chart is elaborated in Figure 8. The wake-up mode with self-hibernation, digital hysteresis, and wake-up interrupt (WU-I) is the default flow in the wake-up period for ensuring WuRx reliability and accuracy. On power up, FSMC is in a wake-up self-hibernation (WUSH) state. The proposed self-hibernation technique reduces the power consumption of IDC and OSC significantly in the non-wake-up interval by configuring OSC to its lowest self-hibernation frequency, *f_SH_*. The dynamic power of a circuit is directly proportional to its operating frequency, as shown in (2). If the frequency reduces, the power consumption also reduces. When IDC detects high assertion on the *wusig* signal during self-hibernation, it configures OSC to its normal wake-up frequency, *f_WU_*, and waits for OSC settling in the WUSH state. The *f_WU_* is a much higher frequency than *f_SH_* for achieving higher wake-up signal measuring accuracy. The controller starts sensing a wake-up signal in the wake-up signal sense (WUSS) state. The SPEG detects *wusig* every rising edge and generates a *wu_pe* pulse signal which is sensed in the WUSS state. The controller enables CWDT by asserting a high *wdt_en* signal. The CWDT provides a self-recovery mechanism for FSMC and it is enabled to avoid uncertain situations and improve IDC reliability. If *WU_SIG* is a noise pulse or glitch, it is identified and filtered out at this stage and FSMC moves back to WUSH for self-hibernation. After the sensing stage, the controller clears CWDT by asserting a high *wdt_clr* signal for one clock cycle and moves to next state. The IDC verifies the *WU_SIG* signal in the wake-up signal assurance (WUSA) state. The AFMU evaluates *WU_SIG* each cycle and confirms if its frequency *f_SIG_* is in a configured range. The integrated configurable digital hysteresis technique accomplishes this task and accommodates wake-up frequency variations to improve reliability. The *WU_SIG* is a valid signal if its frequency fulfills the following condition described in (4) as follows:

(4) $$f_{MOD.MIN}\geq f_{SIG}\leq f_{MOD.MAX},$$

where *f_MOD.MIN_* and *f_MOD.MAX_* are the lower and upper limits of the valid WuRx signal modulation frequencies. These limits are configurable by parameters *N_NFX_* and *N_XFN_*, respectively, and are described as follows in (5):

(5a) $$f_{MOD.MIN}=\frac{f_{WU}}{N_{XFM}},$$

(5b) $$f_{MOD.MAX}=\frac{f_{WU}}{N_{NFX}}.$$

The configurable successive number of wake-up signal cycles, *WU_N_*, in the allowed frequency bandwidth are ensured in the WUSA state. If the *WU_SIG* WuRx signal frequency is not in the allowed range as described in (4) or the signal cycles are less than *WU_N_*, then it means the signal is not a valid wake-up signal and a main transceiver must remain off to save power. The false and poor wake-up signals are identified and filtered out in the WUSA state by AFMU and CWDT successfully. If a non-wake-up signal is identified in this state, the controller moves back to a self-hibernation state. After sensing and assurance, the wake-up interrupt *WU_INT* is initiated for configurable *T_HOLD_* duration in the wake-up interrupt generation (WUIG) state. The interrupt hold duration is defined as follows in (6):

(6) $$T_{HOLD}=\frac{N_{HOLD}}{f_{WU}},$$

where *N_HOLD_* is the configurable parameter for defining wake-up interrupt hold duration. After the interrupt generation, FSMC remains silent for the *T_SILENT_* interval in the wake-up silent (WUS) state and moves back to the WUSH state. The WUS state prohibits the WuRx to detect the current wake-up signal again if *WU_N_* and *T_HOLD_* parameters are configured to smaller values. The silent interval is programmable from parameter *N_SILENT_* according to (7) as follows:

(7) $$T_{SILENT}=\frac{N_{SILENT}}{f_{WU}}.$$

If wake-up monitoring (WU-M) is enabled by signal *monitor_ctrl,* then the FSMC moves to the wake-up interrupt enable (WUIE) state after confirming *WU_N_* wake-up signal cycles in the WUSA state. It asserts *WU_INT* high and moves to the wake-up monitoring (WUSM) state. The controller continuously evaluates *WU_SIG* for the presence of a valid wake-up signal and it additionally provides *WU_INT* for the entire duration of the wake-up signal instead of *T_HOLD_*. It gives more space to MCU to detect interrupt and trigger an acknowledge signal to RSE at the end of the wake-up signal. The proposed WUM guarantees to pass only a valid wake-up signal and it definitely identifies and filters out all non-wake-up signals. It ensures the accuracy and reliability of WuRx. The IDC turns on a main heavy powered transceiver at OBU only with a valid wake-up request from RSE. 

#### 3.3.2. Wake-On Mode

The pseudo-synchronous wake-on mode (WOM) enhances overall reliability of transceiver in case of an issue in the main WuRx path. When WOM is enabled, the IDC configures OSC to desire frequency *f_WO_* based on configured parameters in the wake-on oscillator configuration (WOOC) state, as shown in Figure 8. The wake-on interrupt *T_WOI_* and wake-on sleep *T_WOS_* intervals are computed according to (8) and (9), respectively, as follows:

(8) $$T_{WOI}=\frac{N_{WOI}}{f_{WO}},$$

(9) $$T_{WOS}=\frac{N_{WOS}}{f_{WO}}.$$

After frequency configuration and OSC settling, the controller moves to a wake-on interrupt (WOI) state. In this state, *WO_INT* is asserted high for the *T_WOI_* duration and MCU turns on the main receiver for intercepting any communication request from RSE. After the *T_WOI_* interval, FSMC jumps to a wake-on sleep (WOS) state. The *WO_INT* is asserted low and MCU turns off the main receiver for the programmed *T_WOS_* duration. After a sleep interval, the controller moves back to the WOI state and periodically generates configurable wake-on interrupt *WO_INT* for receiver. 

## 4. Ultra-Low Power Configurable RC Oscillator

**Figure 9:**
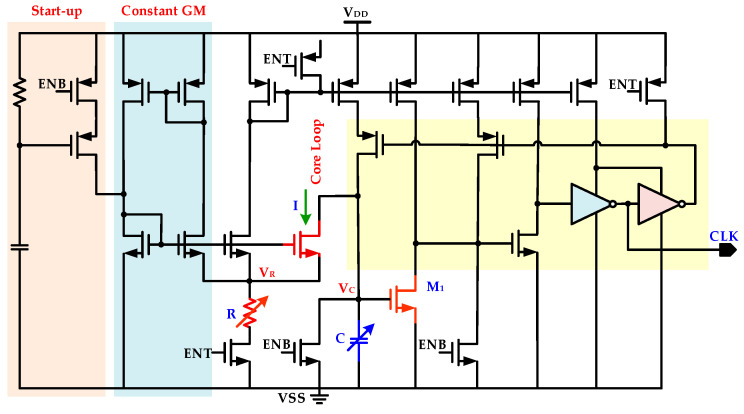
Ultra-low power configurable range communication (RC) oscillator.

In the proposed WuRx, an ultra-low power configurable RC oscillator is integrated, which is the clock source for IDC. The RC oscillator structure is adopted rather than the crystal oscillator due to its low cost, less power consumption, fast start-up interval, and easy on-chip integration [27]. The fabricated oscillator has a wide controllable tuning range, *f_CLK.MIN_*~*f_CLK.MAX_*, and IDC configures it for different frequencies in wake-up, self-hibernation, and wake-on mode by controlling its capacitance values. For ultra-low power applications, the circuits are preferred to be operated in a weak inversion region, also known as the sub-threshold region [28,29]. Therefore, the oscillator is designed to operate in a sub-threshold region. Figure 9 shows the ultra-low power RC oscillator [27]. The configurability for various frequencies is achieved by altering resistance *R* and capacitance *C* values from IDC. This oscillator is composed of current reference, start-up, capacitor charge/discharge sensing circuits, and a frequency generation part. The current source or sink circuit is preferable to function in a weak inversion region for low power operation. The MOS transistor is operating in a sub-threshold region when the drain current *I_D_* flows and the gate to source voltage *V_GS_* is less than the threshold voltage *V_TH_*. The diffusion current between source and drain mainly contributes to this current. The start-up circuit prohibits self-biased circuits to work at a zero biasing point. To enhance current sink or source output resistance, the current mirrors in a cascade structure are used. This generated current is mirrored by the current mirror and fed to capacitor, hysteresis controller *M*_1_, and current-starved invertors for clock generation. The drain current *I* charges the capacitor *C*, and when *V_C_* becomes equal to hysteresis controlling transistor *M_1_ V_TH_* value, the *M*_1_ turns on. For clock frequency generator circuit, the voltage *V_C_* across capacitor *C* linearly increases with the increase in current *I* when constant current flows into the capacitor, as described in (10) as follows:

(10) $$\Delta V_{C}=\frac{I\Delta t}{C}.$$

The transistor *M*_1_ logical *V_TH_* also controls capacitor voltage. Hence, the capacitor *C* charging and discharging duration is controlled by current *I* and transistor *M*_1_, and a triangular voltage waveform is generated for the capacitor. From (10), single charging or discharging cycle time Δ*t* is given in (11):

(11) $$\Delta t=\frac{C\Delta V}{I}.$$

The current mirror builds a constant current source as a current generator part. The voltage *V_R_* is always stabilized by the feedback path. Hence, according to Ohm’s law, the resistance *R* decides the amount of current. Thus, from (11), the generated clock period *T_CLK_* is described in (12) as follows:

(12) $$T_{CLK}=2RC\frac{\Delta V_{C}}{V_{R}}.$$

The RC oscillator output frequency *f_CLK_* is given in (13) as follows:

(13) $$f_{CLK}=\frac{1}{T_{CLK}}=\frac{1}{2RC}\cdot \frac{V_{R}}{\Delta V_{C}}.$$

The capacitance *C* is designed as unit weighted capacitor bank, and IDC configures this capacitor back at different values for generating frequencies *f_WU_*, *f_SH_*, and *f_WO_* for the wake-up mode, self-hibernation, and wake-on mode, respectively. 

## 5. RF Front End and Baseband Processing

**Figure 10:**
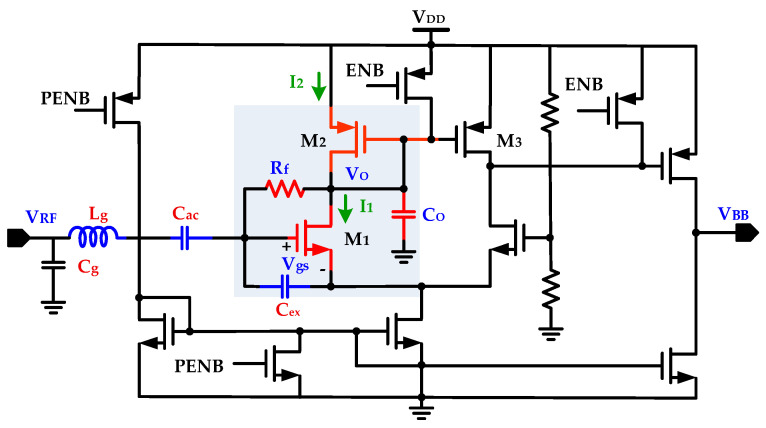
RF envelope detector with internal matching and self-biasing feedback resistor.

The RF front end in the proposed WuRx is composed of a high sensitivity RF envelope detector with an embedded internal matching network. The RFED-based approach is most common for designing a WuRx circuit for its low current consumption. With this scheme, the requirement of a LO generation for frequency down-conversion and RF amplification is also eliminated at the cost of reduced sensitivity. The internal matching improves sensitivity of the circuit. The RFED is the key building block in the WuRx circuit for interfacing with an antenna through a matching network and generating a baseband output signal by down-converting the input amplitude-modulated RF signal. Figure 10 illustrates the proposed RF envelope detector circuit with internal matching network [18,30]. The circuit is mainly composed of nonlinear transistor element *M*_1_, input signal DC blocking capacitor *C_ac_*, self-biasing feedback resistor *R_f_*, impedance matching network with gate inductor *L_g_*, and excess capacitor *C_ex_*. The impedance matching network provides passive voltage amplification. With large self-biasing resistance, when RF input signal is not present, the *M*_1_ gate to source voltage *V_gs_* is closed to transistor threshold voltage *V_TH_* with a very negligible biasing current. In this way, the limited sensitivity issue due to *V_TH_* loss is compensated. The *R_f_* is designed with a pseudo-resistor for large resistance with minimized parasitic capacitance and a small area. It perfectly isolates output voltage *V*_0_ from RF input signal *V_RF_* and prevents envelope detector loading. When *V_RF_* is applied, the drain current *I*_1_ exponentially increases while the biasing current supplied from *M*_2_ is almost constant. 

The capacitor *C_O_* discharges and this discharge current decreases *V_O_* until *I*_1_ and *I*_2_ become almost equal. This generates a 180° phase shift between *V_RF_* and *V_O_*. The convergence gain *G_C_*, defined as the ratio of output baseband signal voltage amplitude to the RF input signal voltage amplitude, is evaluated as follows in (14):

(14) $$G_{C}=\frac{1}{4}\frac{Q^{2}I_{1}}{{\left(nU_{T}\right)}^{2}}r_{0},$$

where *Q* is quality factor of the matching network, *n* is sub-threshold slope factor, *U_T_* is thermal voltage, and *r*_0_ is intrinsic output impedance. 

**Figure 11:**
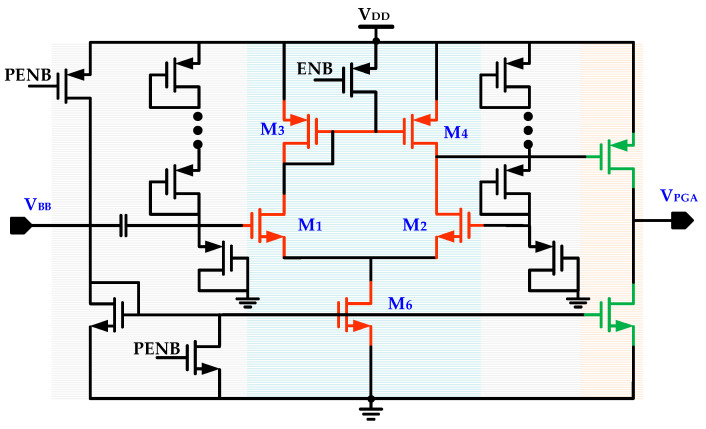
Programmable gain amplifier.

The baseband signal *V_BB_* produced by the RF envelope detector is subsequently processed for enhancing amplitude by a programmable gain baseband amplifier and a comparator before it is fed to IDC for digital processing for generating an interrupt signal. Figure 11 shows the PGA circuit that is a baseband amplification stage. It has high input impedance and its gain is configurable from external MCU. It provides flexibility for improving gain and amplifies the baseband signal *V_BB_* significantly.

**Figure 12:**
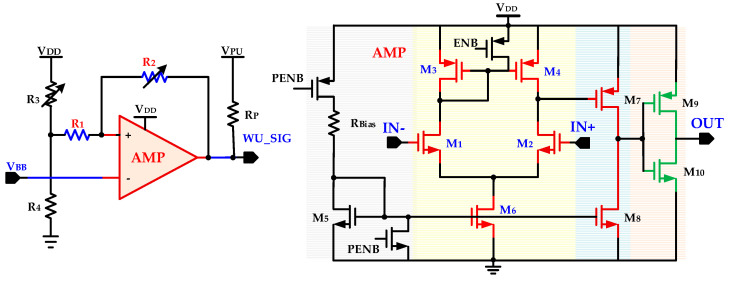
Hysteresis comparator with positive feedback and amplifier.

The hysteresis comparator, shown in Figure 12, is the final stage of baseband processing to generate a digital signal for IDC processing. It is composed of a positive feedback circuit with an amplifier, AMP. A two stage amplifier with output inverter [31] is used as a low power CMOS amplifier. With feedback resistor *R*_2_, hysteresis upper and lower threshold values are configured for eliminating multiple transitions caused by noise. The three stage amplifier is composed of a differential amplifier, common source amplifier, and an output inverter. The analog differential input signals *IN−* and *IN+* are applied at differential pair *M*_1_ and *M*_2_. The differential pair transistor width is increased to reduce input offset voltage and increase gain. To minimize the propagation delay and reduce common source transistor *M*_7_ gate parasitic capacitance, the *M*_7_ is designed with a small size. The final inverter stage also enhances gain and improves the comparator slew rate. The final digital wake-up comparator output signal *WU_SIG* is fed to IDC for digital processing. The RF front end and analog baseband processing processes all signals and it does not filter or remove any non-wake-up signal. The IDC differentiates between actual wake-up signals and unwanted signals.

## 6. Experimental Results

**Figure 13:**
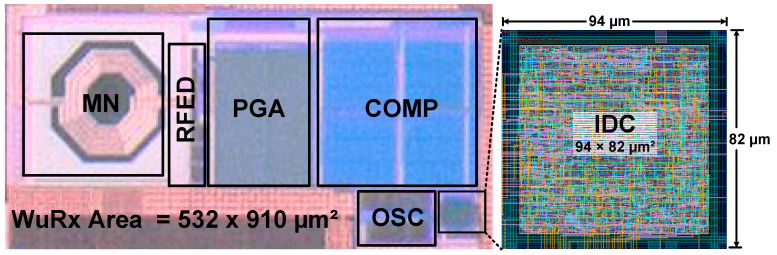
WuRx chip microphotograph and magnified IDC layout.

**Figure 14:**
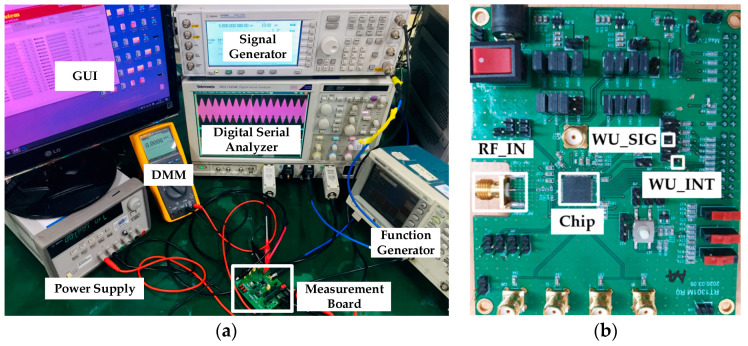
WuRx measurement: (**a**) experimental lab setup; (**b**) measurement board with chip.

The presented WuRx is integrated in a DSRC transceiver for ETCS applications. It is fabricated with a 130 nm CMOS process. Figure 13 shows the microphotograph of WuRx and magnified IDC layout. The WuRx occupied chip area is 532 × 910 µm^2^ of which IDC takes only 94 × 82 µm^2^. The WuRx is measured extensively to ensure its reliability and accuracy. The experimental lab environment is captured in Figure 14a and the measurement board with the fabricated DSRC transceiver chip is depicted in Figure 14b. The board is powered up from the Agilent^®^ DC Power Supply with 5 V and the on-chip low dropout regulator (LDO) generates 0.9 V for the WuRx circuit, which is measured at the output pin with a digital multi-meter (DMM). The lower supply voltage is used to minimize the power consumption and all blocks, and correct operation is verified at a supply voltage of 0.9 V. The OOK baseband wake-up signal is generated from the Tektronix^®^ AFG3101 Function Generator and modulated at 5.8 GHz with the Agilent^®^ E4438C Signal Generator. This modulated RF signal is fed at a *RF_IN* SMA input connector on the board and after passing through external pi-matching network, package pin, and die PAD, it enters the WuRx circuit. The comparator output *WU_SIG* and wake-up interrupt *WK_INT* are plotted on a Tektronix^®^ DSA71254C Digital Serial Analyzer. Different parameters and configurations are programmed through SPI and the graphical user interface (GUI) running on computer.

The IDC performance is summarized in Table 1. The proposed digital controller is fully synthesizable. With an area of 0.007 mm^2^ and a 34.62 nW power consumption, it not only ensures WuRx reliability and accuracy but also replaces complex and power hungry analog blocks such as BPF and ADC. The configurability, operating modes, digital hysteresis, and self-hibernation features prove its sublimity. The power consumption with and without self-hibernation for IDC and OSC and its effect on overall WuRx power performance is summarized in Table 2. Since the DSRC communication between RSE and OBU lasts for a very short interval and the OBU is in sleep mode most of the time, self-hibernation by voltage and frequency scaling has a significant positive impact on battery performance. The performance comparison of the proposed WuRx with the existing designs is listed in Table 3. The wake-up circuits in [8,24] integrate the complex BPF and [9] use the frequency detector (FD) circuit as its interface output stage without ensuring reliability and filtering of non-wake-up signals. The proposed WuRx architecture incorporates a fully synthesizable intelligent controller, which is not only area and power efficient but it also ensures unwanted signals filtering, guarantees WuRx reliability, and improves battery performance. The measurement results report an almost identical sensitivity of −46 dBm and a power consumption of only 2.48 µW.

**Figure 15:**
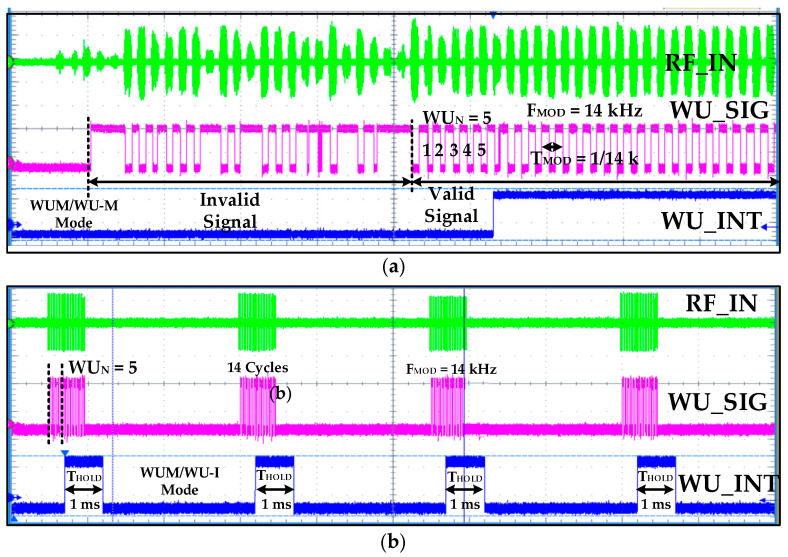
WuRx measurement result: (**a**) initially, invalid random sequence and then valid wake-up signal; (**b**) wake-up valid signal burst for robustness testing.

The WuRx accuracy and reliability is verified by applying various RF-modulated valid and invalid signals with different amplitudes and frequencies at the *RF_IN* input. When the input signal is valid, meaning its amplitude is greater than the sensitivity and its frequency is in a configured hysteresis range, the WuRx gives out confirmed wake-up interrupt signal. In the measurement results in Figure 15a, initially random, invalid OOK sequence, modulated at 5.8 GHz, with a sensitivity of −46 dBm is applied at the *RF_IN* input. The baseband signal is successfully recovered by RFED and the digital *WU_SIG* from COMP is fed to IDC. As it is clear from the results, the IDC identifies this invalid sequence and does not generate a confirmation signal. Later, the valid wake-up signal is ensured and verified by IDC. If IDC is not used at the comparator output, then WuRx reliability degrades as the main power hungry receiver is turned on, even with a non-wake-up signal. The consecutive burst of valid wake-up signals, as shown in Figure 15b, with exactly 14 clock cycles at 14 kHz is applied for proving proposed WuRx robustness, accuracy, and reliability. For each time, *WU_INT* is generated for approximately 1 ms after confirming five clock cycles (*WU_N_* = 5) and IDC returns to its ideal state for the next wake-up signal sensing.

**Figure 16:**
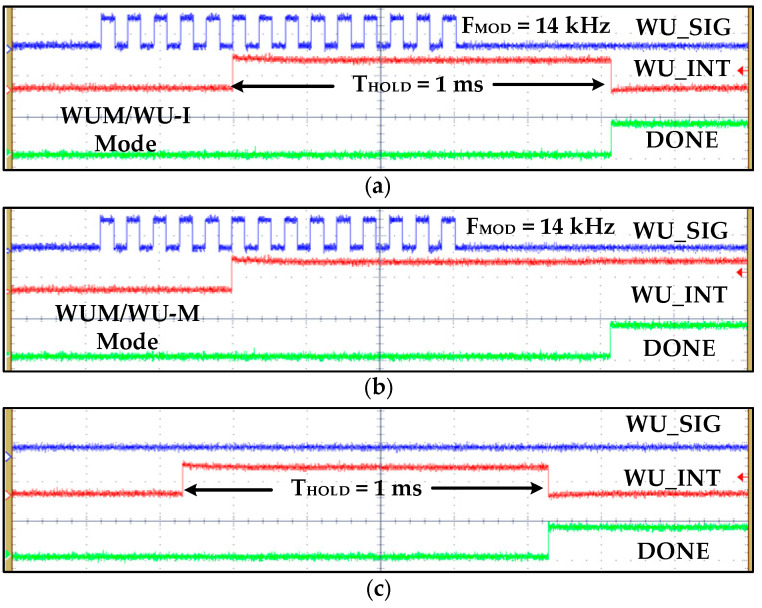
WuRx measurement results for different IDC wake-up modes with valid signals: (**a**) wake-up interrupt (WU-I); (**b**) wake-up monitoring (WU-M); (**c**) self-test with WU-I mode.

Figure 16 shows different WUM measurement results for various scenarios. In this measurement, *WU_N_* is set to 5 and digital hysteresis, watch-dog timer, and *T_HOLD_* are configured to 11~18 kHz, 142.8 µs, and 1 ms, respectively. The OSC is configured for wake-up and self-hibernation frequencies of 140 kHz and 14 kHz, respectively. Figure 16a,b show WUM with WU-I and WU-M configurations, respectively, in which the *WU_SIG* frequency is 14 kHz. The *WU_INT* is generated after sensing and confirming five successive *WU_SIG* clock cycles. In normal WUM, IDC and OSC current consumption from a 0.9 V supply is 38.47 nA and 214 nA, which reduces to 9.7 nA and 107 nA in self-hibernation, respectively. The self-test is measured in Figure 16c in which the *st_sig* signal of 14 kHz is generated by STPG. 

**Figure 17:**
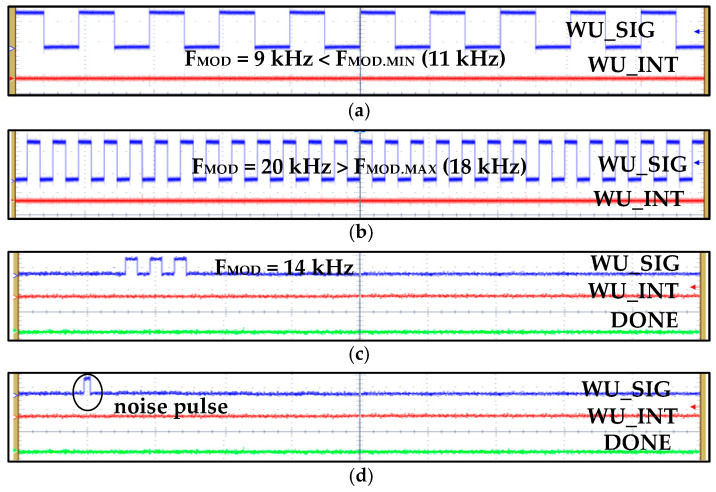
WuRx measurement results for invalid signals and IDC identification and filtering: (**a**) invalid wake-up signal with modulation frequency of 9 KHz, which is less than the minimum hysteresis configured limit of 11 kHz; (**b**) invalid wake-up signal with modulation frequency of 20 KHz, which is greater than the maximum hysteresis configured limit of 18 kHz; (**c**) poor wake-up signal with valid modulation frequency but less number of cycles than the configured value of 5; (**d**) invalid signal of noise pulse.

Figure 17 summarizes WuRx measurement results with false, poor, invalid signals. Figure 17a,b shows results when the *WU_SIG* frequency is out of the configured hysteresis range (11 kHz~18 kHz in this case) and identifies false wake-up signals. Poor and false wake-up and noise signals are also perfectly identified and *WU_INT* is not generated. The signal with a valid frequency but insufficient number of cycles (less than *WU_N_* = 5) is identified and filtered accurately by IDC, as shown in Figure 17c. Similarly, noise pulses and glitches in the RF signal are converted to a baseband digital signal and sensed and removed by IDC without generating interrupt and prohibits turning on the power hungry main receiver. If IDC is not integrated, then all these invalid signals are identified as wake-up signals, and as a consequence, power on transceiver falsely and degrading battery performance.

**Figure 18:**
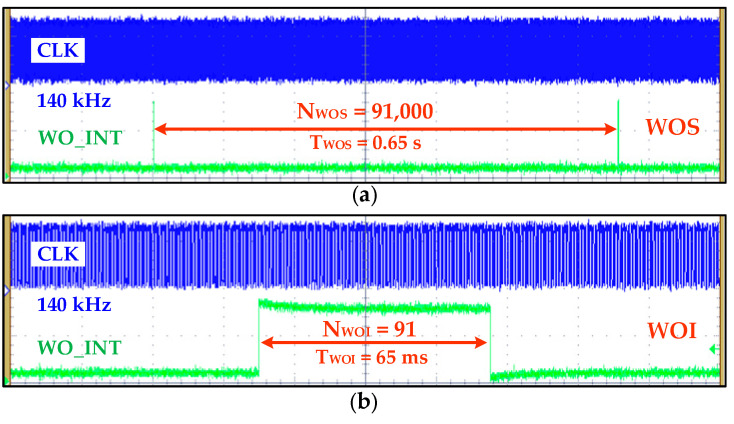
Wake-on mode measurement result: (**a**) Wake-on sleep of 0.65 s duration when *N_WOS_* is configured with value of 91,000; (**b**) wake-on interrupt of 65 ms when NWOI is configured as 91.

The WOM measurement result is depicted in Figure 18 in which *T_WOI_* and *T_WOS_* intervals are set for 65 ms and 0.65 s by configuring *N_WOI_* = 91 and *N_WOS_* = 91,000 according to (8) and (9), respectively.

**Figure 19:**
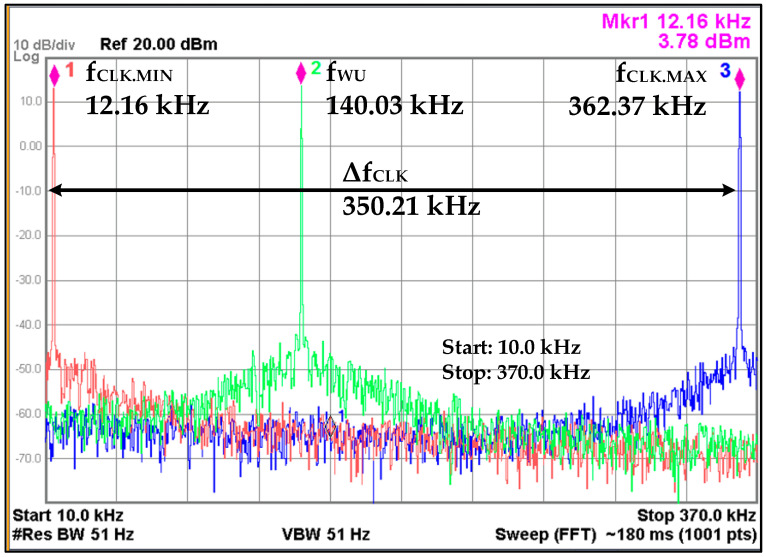
Oscillator (OSC) frequency range and measurement result.

The measured tuning range of OSC is captured in Figure 19. The OSC capacitor *C* is implemented as binary weighted capacitor bank which is controlled from an 8-bit *OSC_CTRL* signal from IDC. The measured *f_CLK.MIN_* and *f_CLK.MAX_* frequencies are 12.16 kHz and 362.37 kHz when *OSC_CTRL* values are all high and all low, respectively, with a total frequency range *Δf_CLK_* of 350.21 kHz. The spectrum also shows the WUM frequency which is configured as approximately 140 kHz. In self-hibernation mode, the OSC frequency *f_SH_* is configured to about 14 kHz. The WOM clock frequency depends on the configured parameters for *T_WOI_* and *T_WOS_* intervals. At *f_WU_* of 140 kHz, it draws 214 nA current from 0.9 V supply which is reduced to almost half in self-hibernation mode. 

**Figure 20:**
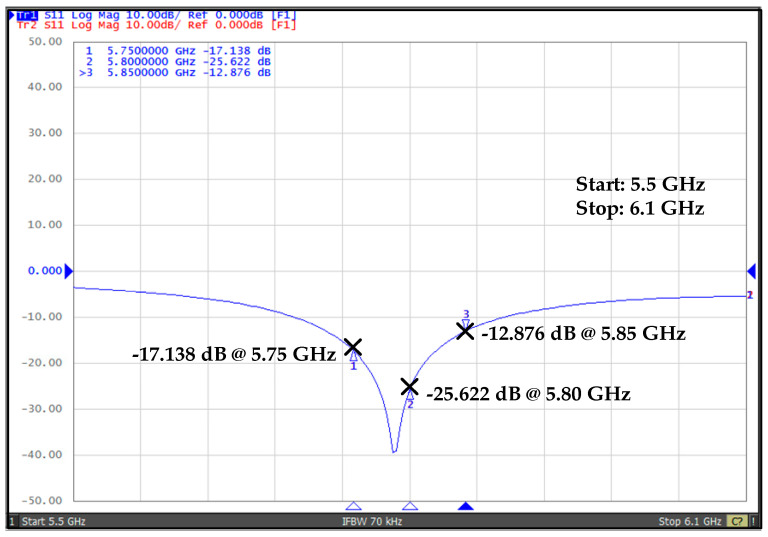
RF external on board impedance matching measurement result.

Figure 20 shows the measured reflection co-efficient, |*S*_11_|, for the proposed WuRx. The measured value of |*S*_11_| at 5.8 GHz is about −25.622 dB, which shows the excellent matching. Moreover, |*S*_11_| values at 5.75 GHz and 5.85 GHz are −17.138 dB and −12.876 dB, respectively.

**Figure 21:**
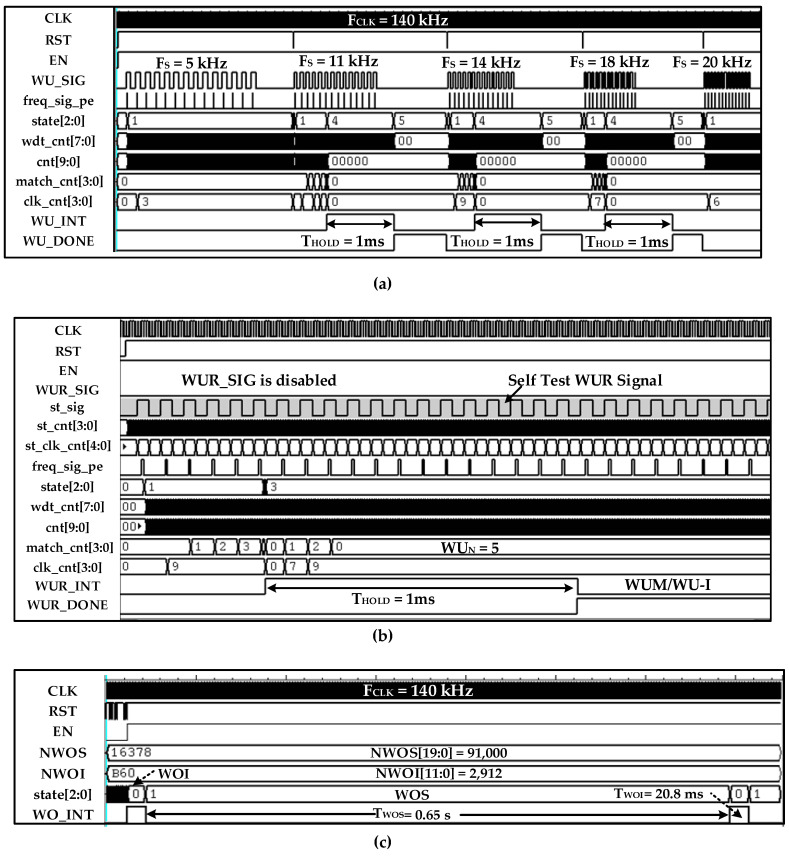
The IDC post place and route (P&R) simulation result: (**a**) WU/WU-I with different wake-up frequencies; (**b**) self-test with WU_I; (**c**) wake-on mode with interrupt and sleep durations of 20.8 ms and 0.65 s.

Figure 21 summarizes the detailed IDC post place and route (P&R) simulation results using the NC-Verilog^®^ tool. The wake-up interrupt mode simulation result is shown in Figure 21a in which *WU_SIG* with different frequencies is applied. It is clear from the simulation results that when the wake-up signal is either less or greater than the configured hysteresis range (11 kHz~18 kHz), it is identified and filtered out without generating interrupt at *WU_INT*. The self-test simulation with one of the configurations is shown in Figure 21b. The STPG generates a variety of valid and invalid signals for ensuring the functional accuracy of IDC. Instead of a baseband digital *WU_SIG* signal, the test wake-up signal *st_sig* is generated internally by STPG. The IDC accurately generates interrupt *WU_INT* after identifying and verifying the signal, as shown in Figure 21b. The self-test enhances the reliability of IDC itself. The WOM simulation is depicted in Figure 21c in which *T_WOI_* and *T_WOS_* intervals are set for 20.8 ms and 0.65 s by configuring *N_WOI_* = 2912 and *N_WOS_* = 91,000 according to (8) and (9), respectively.

## 7. Conclusions

A highly reliable RF WuRx is presented for ETC systems in this article. For improving WuRx reliability and enhancing battery performance, the IDC is proposed as final stage. The IDC also acts as filter and replaces complex and power demanding analog blocks such as BPF, ADC, and FD. With the proposed configurable digital controller, high reliability and accuracy are achieved by sensing and ensuring a successive, configurable number of wake-up signal cycles before enabling power hungry RF transceiver. The presented self-hibernation technique reduces IDC and RC oscillator current consumption during the non-wake-up period and improves battery life. The digital hysteresis accommodates wake-up signal frequency variation and enhances WuRx accuracy. To avoid uncertain conditions during poor and false wake-up, a watch-dog timer for IDC self-recovery is integrated. During wake-up, the digital controller requires 34.62 nW power. In self-hibernation mode, its current reduces from 38.47 nA to 9.7 nA. It is fully synthesizable and needs 809 gates for its implementation in a 130 nm CMOS process with an area of 94 × 82 µm^2^. The WuRx measured power consumption is 2.48 µW, has −46 dBm sensitivity, and a 0.484 mm² chip area. The extensive measurement and verification make the proposed WuRx an ideal solution for a highly reliable DSRC wake-up circuit. 

## Figures and Tables

**Table 1:** IDC performance summary.

Parameter	Value
CMOS process	130 nm
Occupied area	0.0077 mm^2^
Gate count	809
Supply voltage	0.9 V
Current consumption ^1^	38.47/9.7 nA
Power consumption ^1^	34.62/8.73 nW
Wake-up frequency	1–140 kHz
Configurable architecture	Yes
Reliability and accuracy	Digital hysteresis, BIST, WDT
Operating Modes	WUM (WU-I, WU-M), WOM

^1^ Without and with self-hibernation.

**Table 2:** Power consumption summary with and without self-hibernation.

Block	Without Self-Hibernation		With Self-Hibernation	
	Current	Power	Current	Power
Intelligent digital controller	38.47 nA	34.62 nW	9.7 nA	8.73 nW
RC oscillator	214 nA	192.6 nA	107 nA	96.3 nW
Total WuRx	2.75 µA	2.48 µW	2.62 µA	2.36 µW

**Table 3:** WuRx performance comparison.

Parameter	[8]	[9]	[24]	This Work
CMOS process (nm)	130	180	130	130
Wake-up frequency (kHz)	14	7~42	14	1–140 ^1^
Operating frequency (GHz)	5.8	5.8	5.8	5.8
Sensitivity (dBm)	−44	−47	−45	−46
Power consumption (µW)	36	3.8	45	2.48 *
Chip area (mm^2^)	-	-	-	0.484
Interface	BPF	FD	BPF	IDC

^1^ Configurable for any specific range. * Without self-hibernation.
